# Effectiveness of Nurse-Led Digital Health Interventions on Symptom Management and Quality of Life in Cancer Patients Undergoing Systemic Therapy: A Systematic Review of Randomized Controlled Trials

**DOI:** 10.3390/curroncol33070386

**Published:** 2026-06-25

**Authors:** Omar Alqaisi, Safia Darwish, Faten Harb, Melinda Hysenaj, Lorent Sijarina, Patricia Tai

**Affiliations:** 1 Nursing Department, Al-Zaytoonah University, Airport Street, Amman 11733, Jordan; omaralqaisi119@gmail.com; 2 Administration Department, Faculty of Nursing, Menoufia University, Menoufia 32511, Egypt; s.darwish@zuj.edu.jo; 3 Nursing Department, Faculty of Nursing, Al-Zaytoonah University of Jordan, Amman 11733, Jordan; 4 Department of Allied Medical Professions—Associated Nursing Program, Technical College-Middle East University, Amman 11831, Jordan; f.harb@meu.edu.jo; 5 Faculty of Medicine, University of Prishtina, 1000 Prishtina, Kosovo; melindahysenaj@gmail.com (M.H.); lorentsijarina87@gmail.com (L.S.); 6 Department of Oncology, University of Saskatchewan, 105 Administration Place, Saskatoon, SK S7N 5A2, Canada

**Keywords:** digital health, telehealth, nurse-led intervention, symptom management, patient-reported outcomes, health-related quality of life, cancer, systemic therapy, randomized controlled trial

## Abstract

Cancer patients receiving systemic therapy experience substantial treatment-related symptoms. Nurse-led digital health interventions, e.g., interactive voice response systems, web platforms, mobile apps, and telehealth, have emerged as strategies to strengthen supportive care. To evaluate its effectiveness, this systematic review summarizes evidence exclusively from randomized controlled trials (RCTs). Following Preferred Reporting Items for Systematic Reviews and Meta-Analyses (PRISMA) 2020 guidelines, four databases were searched from inception to January 2025 for eligible RCTs involving adults undergoing anticancer therapy; evaluating nurse-led or nurse-co-led interventions using digital or telecommunication technologies; reporting validated symptom or health-related quality of life (HRQoL) outcomes. Risk of bias was assessed. Nine RCTs (N = 3344) met criteria; seven had low risk of bias. Interventions using telephone systems, web portals, mobile apps, or videoconferencing reduced symptom burden and improved HRQoL. The Symptom Care at Home system reduced symptom burden by ~43%, with greatest effects from combined automated monitoring and nurse practitioner follow-up. Additional benefits included improved anxiety, self-efficacy, patient participation, fewer severe toxicities and hospitalization days. In conclusion, nurse-led digital interventions effectively reduce symptom burden and support HRQoL during systemic therapy. Multicomponent models integrating automated monitoring with structured nursing follow-up and decision support appear most beneficial.

## Simple Summary

Cancer patients receiving systemic therapy frequently experience distressing treatment-related symptoms that impair daily functioning and quality of life. Nurse-led digital health interventions, including interactive voice response telephone systems, web-based platforms, mobile applications, and video conferencing, have emerged as scalable strategies to deliver timely, structured symptom monitoring and support between clinic visits. To evaluate the effectiveness of these interventions, we conducted a systematic review restricted exclusively to randomized controlled trials. Following PRISMA 2020 guidelines, four electronic databases were searched from inception through January 2025. Nine randomized controlled trials enrolling 3344 adult cancer patients receiving systemic anticancer therapy met all eligibility criteria. Across the included trials, nurse-led digital interventions consistently reduced symptom burden and preserved or improved health-related quality of life. Additional benefits included reduced anxiety, enhanced self-efficacy, improved patient participation in symptom management, fewer severe treatment toxicities, and reduced hospitalization days. Multicomponent models that integrate automated symptom monitoring with structured nurse practitioner follow-up and clinical decision support demonstrated the largest and most consistent effects. These findings support integrating nurse-led digital health models into routine oncology care and highlight the central role of nurses in translating digital symptom monitoring into meaningful clinical benefit.

## 1. Introduction

Cancer remains one of the leading causes of morbidity and mortality worldwide with millions of new diagnoses annually and a growing survivor population [1,2]. During systemic anticancer therapy, patients frequently experience clusters of distressing symptoms such as fatigue, pain, nausea, and psychological distress that adversely affect daily functioning and health-related quality of life (HRQoL) [3]. These symptoms often fluctuate between clinic visits and, when poorly controlled, are associated with increased unplanned emergency department visits, hospitalizations and treatment disruptions [4]. Consequently, optimizing symptom management and preserving quality of life have become core outcomes in contemporary oncology care [5,6].

Telehealth and broader digital health technologies have rapidly expanded as modalities to support remote cancer care, particularly in the context of the COVID-19 pandemic, which accelerated the adoption of telephone, web-based, and mobile app-based contacts between patients and oncology teams [7,8]. These modalities can facilitate frequent symptom monitoring, timely triage, and tailored self-management support without requiring patients to travel, thereby potentially improving access to care, especially for those who are frail, live in rural areas, or face financial and logistical barriers [9,10].

Nurses are central to telehealth-based cancer care. Nurse-led telephone triage and remote symptom support programs efficiently assess symptom severity, deliver evidence-based self-care guidance, adjust supportive medications with oncologists, and quickly identify patients requiring urgent in-person assessment [4,11]. Randomized trials and meta-analyses demonstrate that nurse-led interventions, both in-person and remote, reduce targeted symptom burdens, strengthen patient self-efficacy, and relieve psychological distress [12,13]. For instance, nurse-led programs significantly cut rates of constipation, insomnia, and financial difficulties, although their effects on global HRQoL and European Organisation for Research and Treatment of Cancer Quality of Life Questionnaire–Core 30 (EORTC QLQ-C30) core domains are inconsistent [12]. Collectively, these findings highlight both the promise and unresolved questions surrounding nurse-led models’ impact on comprehensive quality-of-life outcomes.

In addition to telephone-based telehealth, recent research has investigated digital health platforms such as web portals, interactive voice response systems, and mobile health (mHealth) applications that facilitate real-time patient-reported outcomes (PROs). A landmark randomized trial by Basch et al. [14] demonstrated that web-based symptom monitoring with automated clinician alerts improved symptom control and was associated with enhanced health-related quality of life (HRQoL) and increased survival among patients undergoing chemotherapy. Mooney et al. [8] similarly discovered that an automated telephone patient-reported outcome (PRO) system, coupled with nurse practitioner follow-up upon exceeding symptom thresholds, diminished overall symptom severity during chemotherapy. Systematic reviews of internet- and mHealth-based interventions in oncology have reported beneficial effects on symptom control, distress, self-efficacy, and HRQoL. However, heterogeneity in interventions, populations, and outcome measures limits the ability to draw definitive conclusions [12]. Despite these advances, previous reviews have frequently conflated nurse-led with physician- or psychologist-led interventions, or included survivors beyond the active treatment phase, obscuring the specific contribution of nurse-led digital care during systemic therapy [11,12,15]. Therefore, this systematic review aims to synthesize evidence exclusively from RCTs to evaluate the effectiveness of nurse-led digital health interventions on symptom management and HRQoL among adult cancer patients undergoing systemic therapy.

## 2. Materials and Methods

### 2.1. Study Design and Registration

The systematic review adhered to the Preferred Reporting Items for Systematic Reviews and Meta-Analyses (PRISMA) 2020 guidelines [16]. Registration was completed in the PROSPERO database (PROSPERO registration CRD420261355973) for systematic reviews. Eligibility was restricted to randomized controlled trials (RCTs), including both parallel group and factorial designs. The PRISMA Checklist was enclosed in Supplementary Table S1.

### 2.2. Eligibility Criteria

Study selection was based on predefined inclusion and exclusion criteria structured according to the PICO framework (Population, Intervention, Comparator, Outcomes) [17] (Table 1).

### 2.3. Information Sources and Search Strategy

A systematic electronic search was conducted in January 2025, covering publications from database inception through January 2025, with no date restrictions. The search strategy combined controlled vocabulary (MeSH terms) and free-text keywords, organized around three conceptual domains: (1) cancer and systemic therapy, (2) nurse-led or nursing intervention, and (3) digital health or telehealth modalities. Boolean operators (AND/OR) were used to combine search terms within and across domains. The full search strategy is provided in Supplementary Material Table S2. Additionally, the reference lists of all included studies and relevant systematic reviews were hand-searched to identify eligible studies not captured through database searches. Grey literature and unpublished sources were not included. The search was restricted to English-language publications, reflecting both the review team’s language proficiency and the predominance of English-language RCTs in this field. This restriction represents an acknowledged limitation, as eligible trials published in other languages may not have been captured.

### 2.4. Study Selection

**Figure 1:**
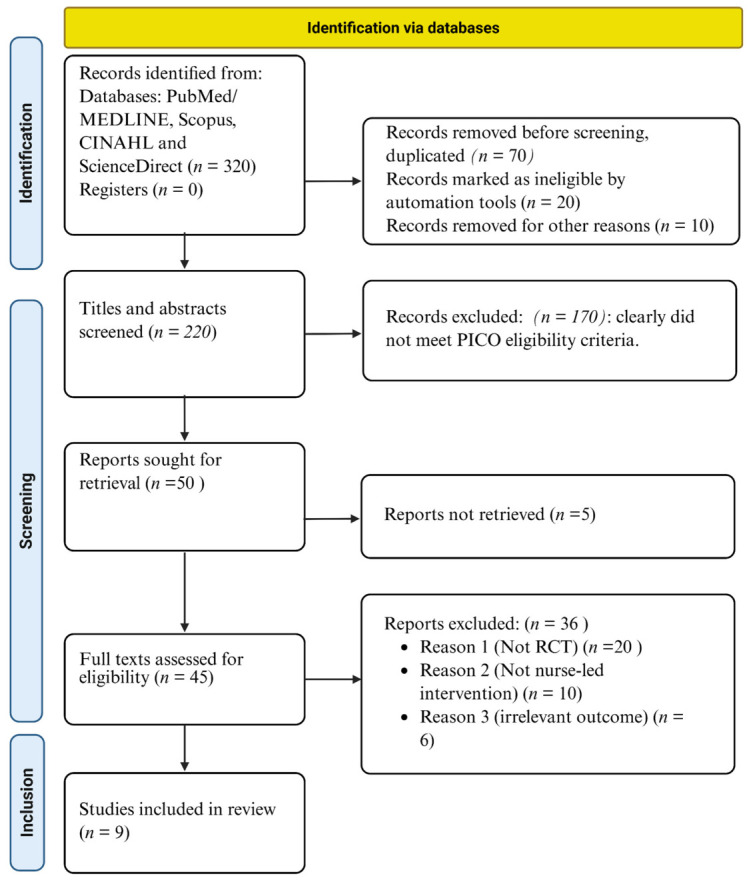
PRISMA 2020 flow diagram. PRISMA: Preferred Reporting Items for Systematic Reviews and Meta-Analyses.

All records identified through database searches were exported to reference management software and deduplicated. Title and abstract screening were independently conducted by two authors (O.A. and S.D.) according to the eligibility criteria. Full-text articles of potentially eligible records were retrieved and independently assessed for final inclusion by the same two authors. Discrepancies at both screening stages were resolved through discussion and, if necessary, by consulting a third author (F.H). The study selection process is presented in the PRISMA 2020 flow diagram (Figure 1).

### 2.5. Data Extraction

The following information was extracted from each included study: authors/years, study design, sample size (*n*), cancer type, intervention.

### 2.6. Risk of Bias Assessment

Two reviewers independently assessed methodological quality and risk of bias for each included RCT. They used the revised Cochrane Risk of Bias tool for randomized trials (RoB 2) [18].

The RoB 2 tool evaluates bias across five domains:

**Randomization process:** Assesses whether the random allocation sequence was properly generated and concealed.

**Deviations from intended interventions:** Examines whether participants and personnel were aware of group assignment and whether deviations from the intended intervention occurred.

**Missing outcome data:** Evaluates the completeness of follow-up and the potential impact of attrition on results.

**Measurement of the outcome:** Assesses the blindness of outcome assessors and the appropriateness of outcome measurement methods.

**Figure 2:**
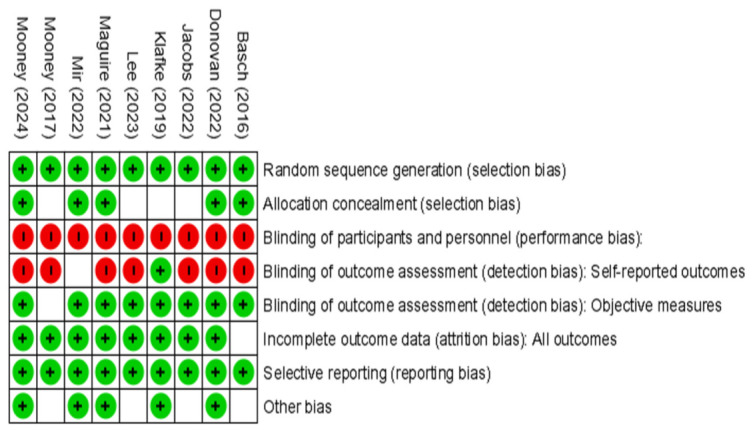
Risk of bias graph of the included randomized controlled trials studies [8,14,19,20,21,22,23,24,25].

**Figure 3:**
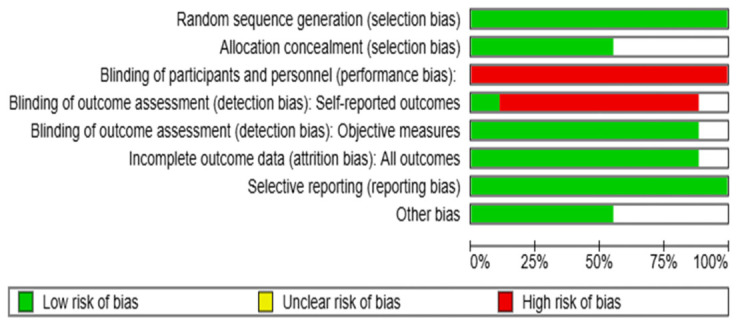
Risk of bias summary of the included randomized controlled trials.

**Selection of the reported result:** Evaluates whether results were selectively reported in a manner inconsistent with the pre-specified protocol. For each domain, a judgement of “Low risk of bias,” “Some concerns,” or “High risk of bias” was assigned based on the tool’s signaling questions and decision algorithm, leading to an overall risk of bias rating per study. Discrepancies in risk-of-bias judgments were resolved through discussion and consensus among the two reviewers; unresolved disagreements were adjudicated by a third reviewer. The risk of bias results is presented as the risk of bias graph (Figure 2) and risk of bias summary (Figure 3).

### 2.7. Synthesis and Data Analysis

Given the anticipated clinical and methodological heterogeneity among the included studies, a meta-analysis was not conducted. Expected differences included intervention components, cancer populations, outcome measures, and follow-up durations. Consequently, a narrative synthesis was employed in accordance with the Synthesis Without Meta-Analysis (SWiM) reporting guidelines.

Since a meta-analysis was not conducted, no pooled effect measures were calculated. Findings are presented as reported in the primary studies, including between-group mean differences, least squares mean differences, odds ratios, and 95% confidence intervals, where available, along with their corresponding *p*-values. All reported effect estimates are taken directly from each trial’s primary analyses.

The decision not to conduct a meta-analysis was based on pre-specified criteria for clinical and methodological heterogeneity. The included studies varied substantially in: (1) patient populations ranging from metastatic solid tumors and recurrent ovarian cancers to patients on adjuvant endocrine therapy and heterogeneous chemotherapy cohorts; (2) intervention platforms and active components including IVR telephone systems, web portals, mobile applications, and videoconferencing, with variable nurse roles (NP-led follow-up, nurse navigator coordination, and nurse-integrated group delivery); (3) primary outcome measures, which included seven distinct validated instruments (EQ-5D, MSAS, SRQ, ePRO-CTCAE scale, BCPT, FACT-G, RDI); and (4) follow-up durations ranging from 8 weeks to 24 months. A subgroup meta-analysis of IVR-based trials was considered; however, the 2024 Mooney et al. factorial design (5 arms) cannot be directly pooled with the 2017 two-arm design [8,19]. To enhance transparency, vote-counting results are presented narratively in the Synthesis section: seven of nine trials (78%) reported statistically significant improvements in their primary symptom or HRQoL outcome, consistent with a predominantly positive direction of effect. A formal effect direction plot was considered and is available from the corresponding author upon request.

### 2.8. Certainty of Evidence

A formal assessment of the certainty of evidence using the Grading of Recommendations Assessment, Development and Evaluation (GRADE) approach was not performed in this review, representing an acknowledged limitation. The clinical and methodological heterogeneity across included studies, including diversity in intervention platforms, cancer populations, outcome instruments, and follow-up durations, precluded the application of GRADE to pooled estimates. Readers should interpret the narrative findings in light of this limitation, recognizing that the overall body of evidence is based predominantly on studies rated as low risk of bias under the Cochrane RoB 2 tool, with seven of nine studies meeting this threshold. Although a formal quantitative GRADE synthesis was precluded by outcome heterogeneity, we conducted a domain-by-domain qualitative GRADE appraisal for the key outcome of symptom burden/severity across the nine included RCTs, following published guidance for GRADE in narrative reviews [26].

(1) Risk of bias: Seven of nine studies were rated as low risk using Cochrane RoB 2; two had some concerns. No downgrading.
(2) Inconsistency: The direction of effect was consistent across seven of nine studies (statistically significant improvement); two studies (Lee et al. and Donovan et al.) reported null findings for symptom burden, although they showed benefits on participation and controllability, respectively. Minor downgrading (−1).
(3) Indirectness: All studies enrolled adult cancer patients receiving systemic therapy with nurse-led digital interventions. No downgrading.
(4) Imprecision: Sample sizes ranged from 100 to 829; effect sizes ranged from d = 0.37 to 0.50. Minor downgrading for variation in precision (−1).
(5) Publication bias: Cannot be excluded given the dominance of positive findings and small study number (*n* = 9). Minor downgrading (−1).

Overall GRADE certainty of evidence for symptom burden outcomes: MODERATE. This indicates that we are moderately confident that nurse-led digital health interventions reduce symptom burden in adult cancer patients on systemic therapy, and that the true effect is likely close to the estimate, although further research may change this conclusion. Readers should interpret these findings accordingly.

### 2.9. Reporting Bias Assessment

Since a formal meta-analysis was not performed, statistical approaches to assess publication bias (e.g., funnel plots and other tests) were not applicable. The potential for selective outcome reporting within each included study was evaluated as part of Domain 5 of the Cochrane RoB 2 tool (Selection of the Reported Result), with all nine studies rated as low risk for selective reporting. The possibility of publication bias at the review level cannot be excluded, given the small number of included studies (*n* = 9); however, a comprehensive four-database search and hand-searching of reference lists were conducted to minimize this risk. The predominance of statistically positive findings across the nine included studies warrants consideration of publication bias as a potential threat to validity. Studies reporting null or negative effects for nurse-led digital interventions may be less likely to be submitted or accepted for publication, introducing a systematic upward bias in the synthesized effect estimates. This concern is amplified by the small number of included studies (*n* = 9), which limits the statistical power to detect asymmetry through funnel plot methods. While our comprehensive four-database search, inclusive date range, and hand-searching of reference lists were designed to minimize retrieval bias, we cannot exclude the possibility that unpublished or negative trials exist. Future systematic reviews with larger numbers of eligible studies should incorporate funnel plot analysis and other tests as formal checks for publication bias.

### 2.10. Reporting Guidelines

The narrative synthesis was organized into the following categories: (1) type of nurse-led digital health platform, (2) primary symptom outcomes, (3) health-related quality of life (HRQoL) outcomes, and (4) secondary outcomes, including anxiety, self-efficacy, and healthcare utilization. Within each category, findings were described and compared across studies, with particular focus on the direction and magnitude of effects, the populations examined, and the outcome measurement instruments used. Clinical heterogeneity was assessed descriptively by comparing participant characteristics, intervention designs, and comparator conditions. Subgroup patterns were explored narratively based on intervention type (reactive versus scheduled nurse follow-up) and cancer population (disease type and treatment phase).

## 3. Results

### 3.1. Study Selection

A total of 420 records were identified across four databases (PubMed/MEDLINE, Scopus, CINAHL, and ScienceDirect). Following removal of duplicates (*n* = 70), records flagged as ineligible by automation tools (*n* = 20), and records excluded for other reasons (*n* = 10), 320 records were retained for title and abstract screening, of which 170 clearly did not meet the predefined eligibility criteria based on population, intervention, comparator, outcomes, and study design. Of the 45 full-text articles assessed for eligibility, 36 were excluded because of non-randomized or single-arm study design (*n* = 20), interventions not led or co-led by nursing professionals (*n* = 10), or absence of a validated symptom or health-related quality of life (HRQoL) outcome measure (*n* = 6). Nine randomized controlled trials (RCTs) fulfilled all inclusion criteria and were included in the narrative synthesis (Figure 1).

### 3.2. Characteristics of Included Studies

The nine included randomized controlled trials (RCTs), published between 2016 and 2024, enrolled a total of 3344 participants across varied settings, cancer types, and intervention modalities. Baseline demographic and clinical characteristics were comparable between intervention and control groups in all studies. Table 2 provides a detailed summary of study characteristics.

The included studies demonstrated substantial variation in technological platforms, intervention components, and follow-up durations. Two studies implemented interactive voice response telephone systems for daily symptom monitoring with nurse practitioner (NP) follow-up [8,19]. The 2024 trial employed a five-group factorial design to ascertain which components of the Symptom Care at Home (SCH) system facilitated symptom reduction. Four trials utilized web-based platforms [14,20,21,22], two employed mobile applications [23,24], and one used videoconferencing as the primary modality [25]. The cancer types studied included advanced solid tumors [14], chemotherapy-treated cancers with curative intent such as breast, colorectal, Hodgkin’s disease, and non-Hodgkin’s lymphoma [22], oral anticancer therapy across all solid tumor types [21], recurrent ovarian cancer [20], breast and gynecological cancers [24,25], and heterogeneous chemotherapy-treated cohorts [8,19,23].

Follow-up durations ranged from 8 weeks [23] primary endpoint to 24 months post enrollment [20]. Most studies monitored participants for the entire duration of one or more chemotherapy courses. The principal outcome in most studies was symptom burden or severity [8,14,19,22].

Other primary endpoints included symptom controllability [20], patient participation in symptom management [23], symptom distress [25], relative dose intensity as a proxy for treatment delivery [21], and global health-related quality of life (HRQoL) [24]. Of the nine included studies, seven were rated as having a low overall risk of bias [8,14,19,20,21,22,24], while two studies [23,25] were rated as having some concerns. Detailed study results of all nine included trials are presented in Table 2.

### 3.3. Assessment of Risk of Bias

The Cochrane Risk of Bias 2 (RoB 2) tool was used to assess risk of bias across five domains: randomization process, deviations from intended interventions, missing outcome data, measurement of the outcome, and selection of the reported result. The overall risk-of-bias ratings are summarized in Table 3. Seven of the nine studies were assessed as having a low overall risk of bias [8,14,19,20,21,22,24]. All studies utilized computer-generated or validated randomization sequences. Allocation concealment was adequate in most studies, employing sealed envelopes, block randomization via web-based systems, or allocation controlled by an independent statistician. Due to the nature of the digital or nursing interventions, participant blinding was not feasible in any study. However, blinding of outcome assessors was implemented in Maguire et al. [22] through statistical team concealment. Several studies also conducted intention-to-treat analyses to address potential bias from non-adherence. Two studies were rated as having some concerns regarding risk of bias: Jacobs et al. [25], a pilot trial in which neither participants nor study staff were blinded to randomization assignment, and Lee et al. [23], a single-center open-label randomized controlled trial where blinding was not possible due to the mobile applications intervention. Attrition rates were generally low across studies. The highest dropout rates occurred in Donovan et al. [20] at 12 weeks (21.1% in Nurse-WRITE (Written Representational Intervention to Ease Symptoms) and 24.1% in enhanced usual care and in Klafke et al. [24] (21% overall during the chemotherapy period). Both studies addressed attrition appropriately using mixed-model analyses or multiple imputation (Table 3).

### 3.4. Narrative Synthesis of Findings

#### 3.4.1. Impact on Symptom Burden and Severity

Seven of the nine included randomized controlled trials (RCTs) assessed symptom burden, severity, or related symptom endpoints as primary outcomes. Most studies reported statistically significant benefits associated with nurse-led digital health interventions. Basch et al. [14] found that among 766 patients receiving outpatient chemotherapy for advanced solid tumors, health-related quality of life (HRQoL; EQ-5D) improved significantly more in the web-based symptom tracking and reporting (STAR) group than in usual care. In particular, 34% of STAR participants reported an improvement in their HRQoL, compared with only 18% of those in usual care. On the other hand, 38% of STAR participants experienced a decline in their HRQoL, compared with 53% of those in usual care (*p* < 0.001). The mean EQ-5D decline was significantly lower in the STAR group (−1.4 points) than in usual care (−7.1 points; *p* < 0.001; effect size = 0.37).

Mooney et al. [19] demonstrated that patients assigned to the Symptom Care at Home (SCH) system (*n* = 180) experienced significantly lower overall symptom severity compared to those receiving enhanced usual care (*n* = 178). The mean treatment impact was −3.59 points in symptom severity (*p* < 0.001), representing an approximate 43% reduction relative to the usual care group. The SCH group saw significant improvements in all 11 individual symptoms, except for diarrhea (*p* < 0.001 to *p* = 0.025). SCH participants also had 67% fewer severe symptom days (odds ratio [OR] = 3.00, 95% confidence interval [CI]: 2.10–4.29, *p* < 0.001) and 39% fewer moderate symptom days (OR = 1.65, 95% CI: 1.24–2.20, *p* < 0.001). Mooney et al. [8] further evaluated the SCH system by randomizing 757 participants into five groups. The complete SCH intervention, which included self-management coaching, nurse practitioner (NP) follow-up, and decision support (Group 5), resulted in the lowest mean symptom burden (mean area under the curve [AUC] = 4.56, 95% CI: 3.90–5.22). This was significantly lower than self-management coaching alone (Group 1: 6.42, difference = 1.86, 95% CI: 1.30–2.41, *p* < 0.001; Group 2: 6.94, difference = 2.38, 95% CI: 1.84–2.92, *p* < 0.001) and NP follow-up without all components (Group 3: 5.13, difference = 0.57, 95% CI: 0.03–1.11, *p* = 0.04; Group 4: 5.22, difference = 0.66, 95% CI: 0.14–1.19, *p* = 0.014). NP follow-up groups consistently outperformed self-management coaching alone (all *p* < 0.001). There were no significant differences between the coaching-only groups (*p* = 0.07) or between the two NP groups (*p* = 0.74), indicating that the combination of all components was necessary for optimal symptom reduction.

Maguire et al. [22], in the multinational eSMART trial (N = 829, 12 European cancer centers), found that the Advanced Symptom Management System (ASyMS) mobile application maintained symptom burden at pre-chemotherapy levels in the intervention group, whereas the control group reported progressive increases from Cycle 1 onward (least squares mean difference = −0.15, 95% CI: −0.19 to −0.12, *p* < 0.001; Cohen’s d = 0.5). Subscale analyses revealed significant between-group differences for the Memorial Symptom Assessment Scale (MSAS) global distress index (−0.21, 95% CI: −0.27 to −0.16, *p* < 0.001), MSAS psychological symptoms (−0.16, 95% CI: −0.23 to −0.10, *p* < 0.001), and MSAS physical symptoms (−0.21, 95% CI: −0.26 to −0.17, *p* < 0.001).

Mir et al. [21] demonstrated in the Cancer Patient Remote Intervention (CAPRI) phase 3 trial (N = 559) that nurse navigator-led digital monitoring significantly improved relative dose intensity (RDI), the primary endpoint. The mean RDI was 93.4% (standard deviation [SD] 25.9) in the CAPRI arm compared to 89.4% (SD 19.1) in the control arm (*p* = 0.04). Grade 3 treatment-related toxicities were significantly lower in the CAPRI arm (27.6% vs. 36.9%, *p* = 0.02), with a significantly lower mean number of toxicity groups (0.4 ± 0.7 vs. 0.7 ± 0.9, *p* = 0.01). Skin disorders were also specifically reduced (3.7% vs. 7.7%, *p* = 0.04).

Donovan et al. [20], in the WRITE Symptoms NRG Oncology trial (N = 497), found that both nurse-guided (Nurse-WRITE) and self-directed (SD-WRITE) web-based interventions significantly improved symptom controllability compared to enhanced usual care (EUC) at both 8 and 12 weeks (group-by-time interaction F = 4.76, *p* < 0.001). Nurse-WRITE participants demonstrated mean controllability increases of 0.234 (standard error [SE] = 0.046, *p* < 0.001) at 8 weeks and 0.215 (SE = 0.052, *p* < 0.001) at 12 weeks. SD-WRITE participants showed similar improvements (0.180, *p* < 0.001 at 8 weeks; 0.162, *p* = 0.002 at 12 weeks). However, no significant group differences were observed for symptom burden (*p* = 0.18) or quality of life (QOL; *p* = 0.24). Lee et al. [23] demonstrated that the electronic Patient-Reported Outcomes version of the Common Terminology Criteria for Adverse Events (ePRO-CTCAE) mobile application significantly improved patient participation in symptom management (8.5 vs. 8.0, *p* = 0.01; group-by-time interaction *p* = 0.02). Consistent benefits were observed across subgroups, including older patients, those with lower digital literacy, and those with lower education. No statistically significant differences were observed in health-related quality of life (HRQoL; *p* = 0.88) or unplanned healthcare visits (*p* = 0.39–0.76), although trends favored the intervention.

Jacobs et al. [25] demonstrated that the Symptom-Targeted Randomized Intervention for Distress and Adherence to Adjuvant Endocrine Therapy (STRIDE) telehealth-based symptom and distress intervention for adjuvant endocrine therapy (AET) was associated with significantly lower symptom distress over 24 weeks (Breast Cancer Prevention Trial [BCPT] slope difference = −1.91, 95% CI: −3.29 to −0.52, *p* = 0.007), including specifically reduced hot flash distress (slope difference = −0.47, *p* = 0.013). The intervention also resulted in significantly better quality of life (QoL; Functional Assessment of Cancer Therapy-Breast [FACT-B] slope difference = 4.66, 95% CI: 2.28–7.05, *p* = 0.001), improved coping skills (Measure of Current Status [MOCS] slope difference = 2.25, *p* = 0.002), and reduced anxiety (Hospital Anxiety and Depression Scale-Anxiety [HADS-A] slope difference = −0.77, *p* = 0.024).

Klafke et al. [24] found that the nurse-led complementary and integrative medicine (CIM) supportive care intervention did not significantly improve global health-related quality of life (HRQoL) at the end of chemotherapy (T3; estimate = −1.04, 95% CI: −4.89 to 2.81, *p* = 0.596). Nonetheless, a notable delayed effect was observed at the 6-month follow-up (T4), with the intervention group showing significantly superior global HRQoL compared to the control group (estimate = 6.643, 95% CI: 1.65–11.64, *p* = 0.010). At T4, the intervention group also demonstrated significantly improved emotional functioning (*p* = 0.007) and reduced fatigue (*p* = 0.027).

#### 3.4.2. Effects on Health-Related Quality of Life

Six studies identified health-related quality of life (HRQoL) as a primary or secondary outcome. Basch et al. [14] demonstrated a clinically meaningful reduction in HRQoL decline in the STAR arm compared to control (−1.4 vs. −7.1 points on EQ-5D, *p* < 0.001). Maguire et al. [22] reported significantly higher FACT-G scores throughout all cycles in the ASyMS group (mean difference = 4.06, 95% CI: 2.65–5.46, *p* < 0.001), with notable improvements in the physical (*p* < 0.001) and functional domains (*p* < 0.001), but not in the emotional or social domains. Jacobs et al. [25] observed significant improvement in FACT-B quality of life over 24 weeks in the STRIDE group (slope difference = 4.66, *p* = 0.001). Klafke et al. [24] reported a significant HRQoL benefit at six months (*p* = 0.010). Donovan et al. [20] found a significant improvement in QoL over time across all study groups (time effect, *p* < 0.001), with no significant group-by-time interaction (*p* = 0.83). Lee et al. [22] reported no significant difference in HRQoL between groups (*p* = 0.88).

#### 3.4.3. Secondary Outcomes: Anxiety, Self-Efficacy, Healthcare Utilization, and Adherence

Anxiety: Maguire et al. [22] reported significantly lower State-Trait Anxiety Inventory—Revised (STAI-R) trait (−1.15, 95% CI: −1.90 to −0.41, *p* = 0.003) and state anxiety (−1.13, 95% CI: −2.06 to −0.20, *p* = 0.02) in the ASyMS group. Jacobs et al. [25] demonstrated significant reductions in anxiety over 24 weeks (HADS-A slope difference = −0.77, *p* = 0.024).

Self-Efficacy: Maguire et al. [22] identified significantly higher self-efficacy in the ASyMS arm (Communication and Attitudinal Self-Efficacy Scale (CASE)-Cancer mean difference = 0.81, 95% CI: 0.19–1.43, *p* = 0.01). Jacobs et al. [25] reported significantly improved coping skills (MOCS, *p* = 0.002) and enhanced self-efficacy for specific AET symptoms, including hot flashes (*p* = 0.019), sleep difficulties (*p* = 0.016), and weight gain (*p* = 0.010).

Healthcare Utilization: Basch et al. [14] found that STAR participants had fewer emergency room visits at one year (34% vs. 41%, *p* = 0.02) and remained on chemotherapy for a longer duration (mean 8.2 vs. 6.3 months, *p* = 0.002). One-year overall survival was significantly higher in the STAR group (75% vs. 69%, *p* = 0.05), with notable quality-adjusted survival benefits (8.7 vs. 8.0 months, *p* = 0.004). Mir et al. [21] reported that CAPRI significantly reduced hospitalization days (mean 2.82 vs. 4.44 days, *p* = 0.02), emergency department visits (15.1% vs. 22.0%, *p* = 0.04), and the proportion of hospitalized patients (22.8% vs. 31.7%, *p* = 0.02). Lee et al. [23] observed non-significant trends toward fewer hospitalizations (33.8% vs. 40.8%, *p* = 0.39) and emergency room visits (19.7% vs. 22.5%, *p* = 0.76).

Treatment Adherence: Mir et al. [21] observed a lower proportion of patients with low adherence to oral anticancer therapy in the CAPRI arm (5.9% vs. 9.8%), although this difference was not statistically significant (*p* = 0.10). Jacobs et al. [25] found no significant differences in adherence to AET, as measured by MARS-5 or MEMS Caps, between STRIDE and MedMon groups.

Supportive Care Needs: Maguire et al. [22] reported significant reductions in the ASyMS group across several SCNS-SF34 domains, including sexuality needs (−1.56, *p* = 0.05), patient care and support needs (−1.74, *p* = 0.03), and physical and daily living needs (−2.80, *p* = 0.01). All results are summarized in Table 4.

## 4. Discussion

This systematic review synthesized evidence from 9 RCTs enrolling 3344 adult cancer patients and found robust, consistent support for the effectiveness of nurse-led digital health interventions in improving symptom management outcomes during systemic anticancer therapy. Across diverse platforms, including interactive voice response, web-based portals, mobile applications, and videoconferencing, and across a wide range of cancer types and treatment regimens, these interventions demonstrated statistically significant and, in several instances, clinically meaningful benefits for symptom burden reduction, HRQoL preservation, anxiety relief, and self-efficacy enhancement. The preponderance of evidence thus supports integrating nurse-led digital health tools into standard oncology practice to reduce the burden of treatment-related symptoms between clinic visits.

The most consistent and robust finding across studies was the reduction in symptom burden and severity. Three studies employing systematic, frequent monitoring protocols [14,19,22] all demonstrated statistically significant and clinically meaningful improvements, with effect sizes ranging from a Cohen’s d of 0.37 [14] to 0.50 [22] and a 43% absolute reduction in overall symptom severity in the Mooney et al. [19] trial. These findings are internally consistent and suggest that the mechanism of benefit is multifactorial: frequent, structured symptom capture facilitates early detection of poorly controlled symptoms; automated or nurse-mediated alerts prompt timely clinical action; and self-management coaching empowers patients to address mild symptoms proactively before escalation. The observed 67% reduction in severe symptom days in the Mooney et al. [19] study demonstrates the clinical importance of these improvements, given that severe symptoms are primary drivers of treatment discontinuation, emergency department visits, and hospitalizations [4].

Particularly illuminating is the five-arm component-deconstruction trial by Mooney et al. [8], which unambiguously demonstrated that no single component of the SCH system is sufficient on its own; rather, integrating automated self-management coaching with nurse practitioner follow-up and decision support produced the greatest reduction in symptom burden. This finding has profound implications for the design of future nurse-led digital interventions, suggesting that neither technological automation alone nor clinician involvement alone is optimal; rather, their synergistic combination is required to achieve maximum clinical benefit. The NP groups were consistently superior to coaching-only groups (*p* < 0.001 for all comparisons), reinforcing the indispensable value of the nursing component in these digital health models.

The Mir et al. [21] CAPRI trial uniquely extended the evidence base to patients receiving oral anticancer agents, a population in which infrequent clinic contacts create significant gaps in symptom surveillance. The nurse navigator-led intervention not only improved relative dose intensity (RDI 93.4% vs. 89.4%, *p* = 0.04) but also significantly reduced grade 3 toxicities (27.6% vs. 36.9%, *p* = 0.02) and hospitalization days (2.82 vs. 4.44 days, *p* = 0.02). These findings are particularly salient given the increasing proportion of systemic anticancer therapy delivered via oral agents, a trend that amplifies the clinical relevance of remote nurse-led monitoring models and demonstrates that such approaches translate into tangible, measurable benefits for both patients and healthcare systems. Notably, the capacity of nurse navigators to independently manage 77.4% of clinical interventions without oncologist referral further underscores the scalability and efficiency of nursing-driven digital care models.

The effects of nurse-led digital interventions on HRQoL were generally positive, though the magnitude and statistical significance of findings varied across studies. The most striking HRQoL results were observed in studies that utilized frequent, high-frequency symptom monitoring combined with active nursing follow-up. Maguire et al. [22] demonstrated a mean FACT-G difference of 4.06 points across all chemotherapy cycles (*p* < 0.001), while Jacobs et al. [25] found a FACT-B slope difference of 4.66 points over 24 weeks (*p* = 0.001). Basch et al. [14] found a clinically meaningful attenuation of EQ-5D decline, showing 5.7 points better than usual care, which further supports the hypothesis that proactive symptom monitoring preserves HRQoL by preventing the accumulation of unmanaged symptom burden.

By contrast, Donovan et al. [20] found no significant group difference in FACT-G QoL (*p* = 0.83), and Lee et al. [23] found no significant HRQoL benefit (*p* = 0.88). These null findings for QoL, however, require contextual interpretation. In the Donovan et al. [20] trial, QOL improved significantly over time across all three groups, including the enhanced usual care group, suggesting that the enhanced usual care protocol itself may have served as an active low-intensity intervention, potentially diluting the between-group effect. In the Lee et al. [23] trial, the study was likely underpowered for HRQoL, as the primary outcome was patient participation in symptom management rather than QoL. The delayed HRQoL benefit observed in Klafke et al. [24] was significant only at 6-month post-chemotherapy follow-up but not at end-of-treatment, suggesting that nurse-led CIM interventions may operate through the strengthening of patient self-management competencies, which require time to manifest as measurable HRQoL improvements. This interpretation is consistent with models of behavioral change in supportive cancer care; wherein psychoeducational and self-care interventions build lasting coping resources that sustain patients through recovery [27].

This review provides novel, clinically important evidence that nurse-led digital interventions confer benefits that extend well beyond symptom severity alone. The eSMART trial [22] is the first large-scale RCT to demonstrate statistically significant improvements in anxiety, as measured by a validated instrument, in a digital remote-monitoring context, with reductions in both trait and state anxiety of approximately 1.1–1.2 points on the STAI-R. These findings are especially noteworthy given that anxiety is among the most prevalent and distressing symptoms experienced by patients on active chemotherapy and is independently associated with worse treatment adherence and QoL. The complementary evidence from Jacobs et al. [25], demonstrating significant HADS-A reductions in the STRIDE group (*p* = 0.024), reinforces the psychological benefits of structured nurse-supported digital care, particularly when interventions incorporate cognitive-behavioral strategies targeting illness representations and symptom-related distress.

Self-efficacy emerged as a consistent secondary benefit across multiple trials. Maguire et al. [22] demonstrated significantly enhanced self-efficacy for cancer communication (CASE-Cancer, *p* = 0.01), while Jacobs et al. [25] found improved self-efficacy for managing specific AET-related symptoms. These findings align with contemporary self-management theory and are consistent with the Chronic Care Model framework underlying the SCH intervention [8], which emphasizes activated, informed patients as a cornerstone of high-quality chronic disease management. Enhanced self-efficacy is not merely an intermediate process outcome; it is a fundamental predictor of treatment adherence, symptom communication, and longer-term health outcomes [28], suggesting that nurse-led digital interventions may produce downstream benefits not fully captured within the relatively short follow-up periods of included trials.

The evidence for reduced healthcare utilization is particularly compelling for clinical and health systems applications. Basch et al. [14] demonstrated a significant 7-percentage-point reduction in ER visits (*p* = 0.02) and the preservation of chemotherapy duration (8.2 vs. 6.3 months, *p* = 0.002), along with a significant survival benefit. Mir et al. [21] documented a clinically important reduction in hospitalization days (1.62 days fewer per patient, *p* = 0.02) and emergency department visits (6.9 percentage points lower, *p* = 0.04). These findings are consistent with the underlying mechanism that proactive, structured symptom management prevents symptoms from escalating to emergency levels, thereby reducing avoidable acute care utilization. The economic implications are substantial: given the high per-episode cost of oncology-related hospitalizations and emergency visits, even modest reductions in these events represent significant value for healthcare systems investing in nurse-led digital monitoring programs.

This review conceptualizes the mechanisms underlying the effectiveness of nurse-led digital health interventions through two complementary theoretical frameworks. First, the Chronic Care Model [29] underpins the approach adopted by Mooney and colleagues, positioning the digital-nurse dyad as a mechanism for shifting cancer symptom care from episodic, reactive, clinic-based encounters toward continuous, proactive, patient-centered monitoring at home [19]. By combining automated monitoring with nurse-mediated clinical action for poorly controlled symptoms, the model aligns treatment intensification precisely with the timing of peak symptom distress, which typically occurs between clinic visits rather than at the point of clinical contact. Second, the Representational Approach [30], which informed the WRITE Symptoms intervention, prioritizes eliciting and reframing patients’ illness representations to motivate and sustain active self-symptom management. The significant improvement in symptom controllability observed in the Donovan et al. [20] trial, but not symptom burden, suggests that representational interventions may be particularly effective at improving patients’ sense of agency over their symptoms, even when objective symptom severity reduction is more challenging to achieve in a population of highly experienced patients with recurrent cancer.

### 4.1. Limitations

Interpreting the findings of this review requires acknowledging several limitations. First, substantial methodological and clinical heterogeneity existed across included studies, encompassing differences in intervention platforms (interactive voice response telephone vs. web-based vs. mobile application vs. videoconferencing), cancer types (mixed solid tumors vs. disease-specific populations), lines of therapy (first-line curative intent vs. recurrent/metastatic), follow-up durations (8 weeks to 24 months), and outcome measures (MSAS, EQ-5D, FACT-G, SRQ, BCPT, custom scales). This heterogeneity precluded meta-analytic pooling and necessitated narrative synthesis. The diversity of platforms and nursing roles, ranging from NP-led follow-up using clinical decision support to nurse navigator-led care coordination to psychologist-delivered group videoconferencing, is classified as nurse-integrated, reflecting the evolving conceptualization of “nurse-led” digital health and introducing uncertainty about which specific nursing functions are the active ingredients of benefit.

Second, most included studies were conducted at a limited number of academic cancer centers with well-resourced oncology practices, predominantly in the United States and Western Europe. The generalizability of findings to community oncology settings, low- and middle-income countries, and populations with limited digital literacy or technology access remains uncertain. While Lee et al. [23] demonstrated consistent benefits across patients with low digital literacy, the study was conducted in a high-mobility smartphone context in South Korea, limiting direct extrapolation.

Third, blinding of participants was not feasible in any included study, introducing potential performance and detection bias. Although most studies employed intention-to-treat analyses and evaluated objective outcomes (symptom severity ratings, healthcare utilization records) alongside patient-reported measures, the open-label nature of all trials should be considered when interpreting self-reported outcomes.

Fourth, the duration of follow-up was limited in several studies, most notably the 8-week primary endpoint in Lee et al. [23] and the 12-week window in the feasibility phase of Jacobs et al. [25], which may have been insufficient to capture the full trajectory of benefit, particularly for interventions targeting self-management competencies or adherence to long-term therapies.

Fifth, none of the included trials measured intervention costs alongside outcomes, precluding cost-effectiveness analysis. Given the substantial investment required to develop, implement, and sustain nurse-led digital monitoring systems, including software infrastructure, nurse training, decision support development, and 24 h alert management, formal health economic evaluations are needed before widespread implementation can be recommended.

### 4.2. Clinical and Research Implications

The converging evidence from this review supports the clinical adoption of multicomponent, nurse-led digital health interventions for patients undergoing systemic cancer therapy. Healthcare institutions should prioritize integrating real-time symptom-monitoring platforms that incorporate both automated self-management support and structured pathways for nursing follow-up, as neither component alone is sufficient to achieve optimal outcomes. The operational model pioneered by the SCH and ASyMS systems provides a scalable template adaptable across clinical settings and cancer types, as nurses respond to algorithm-generated alerts using evidence-based decision support. Broader palliative health literature also includes clinician-led interventions, e.g., Greer et al., although it is beyond the scope of this review which focuses on nurse-led interventions [31].

Future research should address several unresolved questions. Multisite, adequately powered RCTs are needed in community-based and international settings to establish the generalizability of current findings. Head-to-head comparisons of different digital platforms (interactive voice response vs. mobile application vs. web portal) and nursing roles (registered nurse vs. NP vs. advanced practice nurse navigator) would help identify which implementation characteristics confer the greatest benefit. Investigations of the optimal frequency and duration of monitoring, and of patient subgroups who derive the greatest benefit (e.g., by cancer type, treatment intensity, age, or digital literacy), are also warranted. Finally, there is a compelling need for formal economic evaluations alongside future trials to guide resource-allocation decisions in both high-income and resource-limited settings. The component-deconstruction analysis by Mooney et al. [8] provides the strongest evidence to date that multicomponent designs, specifically the combination of automated self-management coaching, nurse practitioner follow-up, and decision support, are superior to any individual component alone (*p* < 0.001 for all pairwise comparisons). Web-based platforms with automated clinician alerting [14] and mobile symptom monitoring with real-time nursing response [22] similarly demonstrated robust benefits. In contrast, self-directed web-based modules without active nursing follow-up [20] and mobile applications with primarily physician-reviewed dashboards [23] produced narrower benefits, limited to symptom controllability and patient participation, respectively. Collectively, these findings indicate that the nursing component, specifically structured, protocol-driven follow-up with decision support, is the active ingredient that transforms digital symptom monitoring into clinically meaningful care. The evidence suggests that patients receiving intravenous chemotherapy for curative-intent solid tumors (breast, colorectal, lymphoma) derive consistent benefits across symptom burden, HRQoL, anxiety, and self-efficacy. Patients on oral anticancer agents, a rapidly growing population, also demonstrated significant gains in dose intensity, reduced toxicity, and fewer hospitalizations through nurse navigator-led monitoring, suggesting that this model is particularly valuable for populations with infrequent clinic contacts. Those with higher baseline symptom burden show the largest HRQoL improvements [14]. Limited evidence is available for patients with hematologic malignancies, pediatric cancers, or those in palliative-only settings, representing gaps for future research. Implementation of nurse-led digital health systems requires investment in software infrastructure, nurse training, clinical decision support development, and dedicated nursing time for alert management. The ASyMS system required approximately 15–20 min of nurse time per alert response, and the SCH system required NP availability during chemotherapy working hours. The CAPRI trial [21] demonstrated that nurse navigators independently managed 77.4% of clinical events without oncologist referral, suggesting that the model is cost-effective in settings with sufficient nursing capacity. Formal cost-effectiveness analyses are urgently needed, as none of the included trials reported health economic data alongside clinical outcomes. All included studies were conducted in high-income countries (United States, United Kingdom, France, Germany, South Korea, Austria, Switzerland), which limits generalizability to low- and middle-income settings where the cancer burden is growing rapidly. Specific challenges in resource-limited settings include: (1) limited smartphone penetration and internet connectivity, particularly in rural areas; (2) insufficient nurse-to-patient ratios that may preclude alert-response protocols; (3) lack of interoperability between digital platforms and existing electronic health records; and (4) limited patient familiarity with digital health tools. Adaptation studies incorporating low-bandwidth solutions (IVR telephone systems remain viable in settings with basic telephone infrastructure) and task-shifting models are recommended.

## 5. Conclusions

This systematic review synthesizes evidence from nine RCTs enrolling 3344 adult cancer patients undergoing systemic therapy. The findings demonstrate that nurse-led digital health interventions delivered via interactive voice response systems, web-based platforms, mobile applications, or videoconferencing consistently reduce symptom burden and preserve health-related quality of life (HRQoL). Beyond symptom and HRQoL benefits, these interventions also reduced anxiety levels and enhanced self-efficacy, as evidenced by significant improvements on validated instruments across multiple independent trials. The most consistent benefits were seen in studies that used frequent, automated symptom monitoring, structured nurse-mediated follow-up, and clinical decision support, underscoring the importance of multicomponent designs in which technological automation and nursing expertise operate synergistically. Nurse-led digital monitoring is also associated with significant reductions in emergency department visits, hospitalization days, and grade 3 toxicities, highlighting its potential to generate substantial value for health systems alongside direct patient benefit. Variability in effect sizes, platform types, and the roles of nursing professionals across studies reflects the field’s heterogeneity and calls for further standardization in the design, delivery, and evaluation of nurse-led digital interventions in oncology. As systemic cancer therapy continues to expand in scale and complexity, the evidence reviewed here supports prioritizing nurse-led digital health as a core element of comprehensive, patient-centered supportive care.

## Figures and Tables

**Table 1:** Eligibility criteria.

Element	Inclusion Criteria	Exclusion Criteria
Population	Adults (≥18 years) with any cancer type receiving systemic anticancer therapy (chemotherapy, targeted therapy, immunotherapy, endocrine therapy)	Pediatric patients; survivors beyond active systemic treatment
Intervention	Nurse-led or nurse co-led digital/telehealth interventions (IVR, web-based portals, mobile applications, videoconferencing, ePRO systems)	Interventions delivered exclusively by non-nursing professionals (physicians, psychologists, pharmacists) with no nursing involvement
Comparator	Usual care, enhanced usual care, or active control without nurse-led digital components	Studies with no comparator group, or with a comparator that includes nurse-led digital components.
Outcomes	Validated symptom measures (e.g., MSAS, CTCAE) and/or HRQoL instruments (e.g., EORTC QLQ-C30, FACT-G, EQ-5D); plus, anxiety, self-efficacy, healthcare utilization, adherence	No validated symptom or HRQoL outcome
Study design	Randomized controlled trials (parallel-group, factorial, or cluster-RCTs)	Observational, quasi-experimental, single-arm, qualitative studies
Language and type	English, full-text peer-reviewed articles	Non-English; conference abstracts, protocols only, grey literature

CTCAE: 30. European Organisation for Research and Treatment of Cancer Quality of Life Questionnaire–Core 30; ePRO: electronic patient reported outcome; EQ-5D: EuroQol 5-Dimension Questionnaire; FACT-G: Functional Assessment of Cancer Therapy–General; HRQoL: health-related quality of life; IVR: interactive voice response; MSAS: Memorial Symptom Assessment Scale; RCT: randomized controlled trials.

**Table 2:** Detailed results of all nine included trials.

Study	Sample Size (Intervention/ Control)	Systemic Therapy	Intervention Platform	Nurse Role	Comparator	Primary Outcome	Follow-up Duration
Basch et al. (2016) [14]	441/325 (N = 766)	Chemotherapy	Web-based (STAR): tablet/kiosk + email alerts	Nurses responded to automated severe-symptom email alerts	Usual care (clinician-discretion monitoring)	HRQoL change from baseline at 6 months (EQ-5D)	Mean 7.4 months (range 0.25–49 months)
Mooney et al. (2017) [19]	180/178 (N = 358)	Chemotherapy (≥3 cycles)	IVR telephone (SCH): daily monitoring + automated coaching + NP follow-up	NPs provided telephone follow-up with decision support for alerted symptoms	Enhanced usual care (daily symptom reporting only)	Overall symptom severity (sum of 11 symptoms, scale 0–10)	Duration of chemotherapy course (mean ~77 days)
Mir et al. (2022) [21]	272/287 (N = 559)	Oral anticancer agents (chemotherapy 39%, targeted therapy 61%)	Web portal/smartphone app (CAPRI) + nurse navigator-led follow-up	Nurse navigators: scheduled follow-up, alert management, clinical decision support	Usual care (oncologist-led clinic visits only)	Relative dose intensity (RDI)	6 months
Donovan et al. (2022) [20]	166 Nurse-WRITE/166 SD-WRITE/165 EUC (N = 497)	Predominantly chemotherapy (85% receiving chemo)	Web-based message board (Nurse-WRITE) or automated web module (SD-WRITE)	Research nurse interventionists: 1:1 asynchronous web-based symptom management sessions	Enhanced usual care (monthly symptom report + online resources)	Symptom controllability (SRQ) at 8 and 12 weeks	12 weeks (with annual follow-up)
Mooney et al. (2024) [8]	143/144/148/155/167 per group (N = 757)	Chemotherapy (≥3 cycles)	IVR telephone (SCH) with component deconstruction across 5 groups	NP follow-up (Groups 3–5) with and without decision support system	Group 1: coaching only (blinded activity tracker); Group 2: coaching + visible tracker	Overall symptom burden (AUC of summed 11-symptom severity)	Duration of chemotherapy (≤6 months; mean 72.6 days)
Maguire et al. (2021) [22]	415/414 (N = 829)	First-line adjuvant or first-time chemotherapy (curative intent)	Mobile app (ASyMS): daily symptom monitoring + 24 h real-time alerts to clinicians	Nurses/clinicians received red/amber alerts and responded per clinical decision support	Standard care (clinic-based monitoring; patient-initiated contact)	Symptom burden (MSAS total score) over 6 cycles	Up to 6 chemotherapy cycles (~6 months)
Lee et al. (2023) [23]	142/71 (N = 213)	Chemotherapy and/or radiotherapy (oral or IV)	Mobile app (ePRO-CTCAE): weekly symptom monitoring + physician dashboard	Research coordinators (nursing staff) educated patients; physicians reviewed dashboard	Usual clinical practice (symptom diary offered)	Patient participation in symptom management (10-item scale) at 8 weeks	8 weeks
Jacobs et al. (2022) [25]	50/50 (N = 100)	Adjuvant endocrine therapy (AET: tamoxifen or aromatase inhibitor)	Videoconferencing (Zoom; STRIDE): 6 weekly group sessions + 2 individual calls	Licensed clinical psychologists/psychology fellows (nurse-integrated delivery)	Medication monitoring only (MedMon): MEMS caps + usual care	Feasibility (enrollment >50%, retention >70%, attendance ≥70%); secondary: symptom distress (BCPT) and QoL (FACT-B) at 12 and 24 weeks	24 weeks
Klafke et al. (2019) [24]	126/125 (N = 251 randomized; *n* = 231 in ITT analysis)	Chemotherapy (curative 85.9%, palliative 14.1%)	Nurse-led CIM (Complementary and Integrative Medicine) in-person + home-based instruction	Trained oncology nurses provided CIM counselling, naturopathic applications, and self-care guidance	Routine supportive care only	Global HRQoL (EORTC QLQ-C30) at end of chemotherapy (T3) and 6-month follow-up (T4)	End of chemotherapy + 6-month follow-up

ASyMS: Advanced Symptom Management System; AET: adjuvant endocrine therapy; AUC: area under the curve; BCPT: Breast Cancer Prevention Trial Symptom Checklist; CAPRI: Cancer Patient Remote Intervention; CIM: Complementary and Integrative Medicine; EORTC QLQ-C30: European Organisation for Research and Treatment of Cancer Quality of Life Questionnaire–Core 30; ePRO-CTCAE: electronic Patient-Reported Outcomes version of the Common Terminology Criteria for Adverse Events; EQ-5D: EuroQol 5-Dimension; EUC: enhanced usual care; FACT-B: Functional Assessment of Cancer Therapy-Breast; HRQoL: health-related quality of life; ITT: intention to treat; IV: intravenous; IVR, interactive voice response; MedMon: medication monitoring only; MEMS, Medication Event Monitoring System; MSAS: Memorial Symptom Assessment Scale; N, number; NP, nurse practitioner; QoL, quality of life; RDI, relative dose intensity; SCH, Symptom Care at Home; SD-WRITE, self-directed WRITE; SRQ, Symptom Representation Questionnaire; STAR: Symptom Tracking and Reporting; STRIDE: Symptom-Targeted Randomized Intervention for Distress and Adherence to AET; WRITE: Written Representational Intervention to Ease Symptoms.

**Table 3:** Risk of Bias Assessment Using Cochrane RoB 2 Tool.

Study	Randomization Process	Deviations from Intervention	Missing Outcome Data	Outcome Measurement	Selective Reporting	Overall Risk
Basch et al. (2016) [14]	Low	Low (unblinded; ITT analysis)	Some concerns (30% missing 6-month HRQoL; sensitivity analyses conducted)	Low	Low	Low
Mooney et al. (2017) [19]	Low	Low (unblinded; ITT analysis)	Low (full ITT; minimal imputation)	Low	Low	Low
Mir et al. (2022) [21]	Low	Low (unblinded; ITT analysis)	Some concerns (51.2% did not complete 6-month follow-up; analyzable population used)	Low	Low	Low
Donovan et al. (2022) [20]	Low	Low (unblinded; ITT via linear mixed models)	Low (missing at random assumption supported)	Low	Low	Low
Mooney et al. (2024) [8]	Low	Low (unblinded; ITT via constrained longitudinal model)	Low (no imputation; all data incorporated)	Low	Low	Low
Maguire et al. (2021) [22]	Low	Low (patients unblinded; evaluator/statistician blinded)	Low (low attrition 8.2–9.2%; mixed model analysis)	Low	Low	Low
Lee et al. (2023) [23]	Low	Some concerns (open-label; physician awareness could introduce attention bias)	Low (ITT; 5/1 withdrew before post-survey)	Some concerns (custom questionnaire; no blinding)	Low	Some concerns
Jacobs et al. (2022) [25]	Low	Some concerns (pilot; no blinding of participants/staff)	Low (92% completed 12-week; 91% 24-week assessment)	Low	Low	Some concerns
Klafke et al. (2019) [24]	Low	Low (unblinded; ITT and PP analyses)	Low (21% dropout; sensitivity analyses with imputation)	Low	Low	Low

ITT: intention-to-treat; PP: per-protocol; RoB 2: Cochrane Risk of Bias Tool version 2.

**Table 4:** Summary of Primary and Key Secondary Outcome Data.

Study	Primary Outcome Measure	Intervention Group Result	Control Group Result	Effect Size/*p*-Value	Key Secondary Outcomes
Basch et al. (2016) [14]	EQ-5D HRQoL change at 6 months	−1.4-point decline	−7.1-point decline	Difference = 5.7 pts; *p* < 0.001; d = 0.37	ER visits: 34% vs. 41% (*p* = 0.02); 1-year survival: 75% vs. 69% (*p* = 0.05); QA survival: 8.7 vs. 8.0 months (*p* = 0.004)
Mooney et al. (2017) [19]	Overall symptom severity (sum 11 symptoms)	Adj. mean 4.795	Adj. mean 8.384	Difference = −3.59; *p* < 0.001	Severe symptom days: 67% fewer (OR 3.00, *p* < 0.001); mild symptom days: 39% more (*p* = 0.016)
Mir et al. (2022) [21]	Relative dose intensity (RDI)	Mean 93.4% (SD 25.9)	Mean 89.4% (SD 19.1)	Difference = 4.0%; *p* = 0.04	Grade 3 toxicities: 27.6% vs. 36.9% (*p* = 0.02); Hospital days: 2.82 vs. 4.44 (*p* = 0.02); PACIC: 2.94 vs. 2.67 (*p* = 0.01)
Donovan et al. (2022) [20]	Symptom controllability (SRQ) at 8–12 weeks	NW: +0.234 at 8w (*p* < 0.001); SDW: +0.180 (*p* < 0.001)	EUC: no change (*p* > 0.05)	Group × time F = 4.76; *p* < 0.001	Symptom burden: no significant group difference (*p* = 0.18); QoL: no significant group difference (*p* = 0.83)
Mooney et al. (2024) [8]	Symptom burden AUC (complete SCH vs. components)	Group 5 (complete SCH): 4.56 (95% CI: 3.90–5.22)	Group 1 (coaching only): 6.42 (95% CI: 5.73–7.10)	Complete vs. coaching: diff = 1.86 (*p* < 0.001)	NP groups superior to coaching alone (all *p* < 0.001); No difference between NP ± DSS (*p* = 0.74)
Maguire et al. (2021) [22]	Total MSAS symptom burden score	Adj. LS mean = 0.36	Adj. LS mean = 0.52	Difference = −0.15 (95% CI: −0.19 to −0.12); *p* < 0.001; d = 0.5	FACT-G: +4.06 (*p* < 0.001); STAI-R trait: −1.15 (*p* = 0.003); CASE-Cancer self-efficacy: +0.81 (*p* = 0.01)
Lee et al. (2023) [23]	Patient participation in symptom management (0–10 scale)	Mean score 8.5 at 8 weeks	Mean score 8.0 at 8 weeks	Difference = 0.5; *p* = 0.01; interaction *p* = 0.02	HRQoL: no significant difference (*p* = 0.88); Hospitalization: 33.8% vs. 40.8% (*p* = 0.39)
Jacobs et al. (2022) [25]	Feasibility (primary); symptom distress (BCPT) & QoL at 12–24 weeks	Slope for BCPT distress: −1.50 (per 12 weeks)	Slope for BCPT distress: −0.40	Difference in slope = −1.91 (95% CI: −3.29 to −0.52); *p* = 0.007	BCPT distress slope difference = −1.91 (*p* = 0.007); FACT-B QoL slope difference = +4.66 (*p* = 0.001); HADS-A anxiety slope difference = −0.77 (*p* = 0.024)
Klafke et al. (2019) [24]	Global HRQoL (EORTC QLQ-C30) at end of chemotherapy (T3) and 6-month follow-up (T4)	T3: 54.5 (SD 20.3); T4: 70.4 (SD 19.8)	T3: 58.3 (SD 19.7); T4: 64.5 (SD 21.1)	T3: *p* = 0.596 (NS); T4: estimate = 6.643 (*p* = 0.010)	Fatigue: *p* = 0.027; Emotional functioning: *p* = 0.007 (both at T4)

AUC: area under the curve; BCPT: Breast Cancer Prevention Trial Symptom Checklist; CASE: Cancer, Communication and Attitudinal Self-Efficacy Scale; DSS: decision support system; EORTC QLQ-C30: European Organisation for Research and Treatment of Cancer Quality of Life Questionnaire–Core 30; EQ-5D: EuroQol 5-Dimension; EUC: enhanced usual care; FACT-B: Functional Assessment of Cancer Therapy–Breast; FACT-G: Functional Assessment of Cancer Therapy–General; HADS-A: Hospital Anxiety and Depression Scale—Anxiety subscale; HRQoL: health-related quality of life; LS: least squares; MOCS: Measure of Current Status; MSAS: Memorial Symptom Assessment Scale; NP: nurse practitioner; NW: Nurse-WRITE; PACIC: Patient Assessment of Chronic Illness Care; QA: quality-adjusted; QoL: quality of life; RCT: randomized controlled trial; SCH: Symptom Care at Home; SDW: SD-WRITE; SRQ: Symptom Representation Questionnaire; STAI-R: State-Trait Anxiety Inventory-Revised.

## Data Availability

No new data were created or analyzed in this study. Data sharing is not applicable to this article.

## Supplementary Materials

The following supporting information can be downloaded at: https://www.mdpi.com/article/10.3390/curroncol33070386/s1, Table S1: PRISMA checklist; Table S2: Search string strategy.

## Abbreviations

The following abbreviations are used in this manuscript:

AET	Adjuvant Endocrine Therapy
ASyMS	Advanced Symptom Management System
AUC	Area Under the Curve
BCPT	Breast Cancer Prevention Trial
CAPRI	Cancer Patient Remote Intervention
CASE-Cancer	Communication and Attitudinal Self-Efficacy Scale for Cancer
CIM	Complementary and Integrative Medicine
CI	Confidence Interval
CTCAE	Common Terminology Criteria for Adverse Events
DSS	Decision Support System
EQ-5D	EuroQol 5-Dimension
eSMART	European multicentre randomised controlled trial
EUC	Enhanced Usual Care
FACT-B	Functional Assessment of Cancer Therapy—Breast
FACT-G	Functional Assessment of Cancer Therapy—General
HADS-A	Hospital Anxiety and Depression Scale—Anxiety
HRQoL	Health-Related Quality of Life
ITT	Intention-to-Treat
IV	Intravenous
IVR	Interactive Voice Response
LS	Least Squares
MARS-5	Medication Adherence Report Scale (5-item)
mHealth	Mobile Health
MEMS	Medication Event Monitoring System
MOCS	Measure of Current Status
MSAS	Memorial Symptom Assessment Scale
NP	Nurse Practitioner
NRG	NRG Oncology Cooperative Group
OR	Odds Ratio
PACIC	Patient Assessment of Chronic Illness Care
PICO	Population, Intervention, Comparator, Outcome
PP	Per-Protocol
PRISMA	Preferred Reporting Items for Systematic Reviews and Meta-Analyses
PRO	Patient-Reported Outcome
PROSPERO	International Prospective Register of Systematic Reviews
QOL	Quality of Life
RCT	Randomized Controlled Trial
RDI	Relative Dose Intensity
RoB 2	Risk of Bias Tool Version 2
SCH	Symptom Care at Home
SCNS-SF34	Supportive Care Needs Survey—Short Form 34
SD	Standard Deviation
SD-WRITE	Self-Directed WRITE (Written Representational Intervention to Ease Symptoms)
SE	Standard Error
SRQ	Symptom Representation Questionnaire
STAR	Symptom Tracking and Reporting
STAI-R	State-Trait Anxiety Inventory—Revised
STRIDE	Symptom-Targeted Randomized Intervention for Distress and Adherence
SWiM	Synthesis Without Meta-Analysis
T3	Timepoint 3 (End of Chemotherapy)
T4	Timepoint 4 (6-Month Follow-Up)
