# Supplementary material for: Effectiveness of Nurse-Led Digital Health Interventions on Symptom Management and Quality of Life in Cancer Patients Undergoing Systemic Therapy: A Systematic Review of Randomized Controlled Trials
Source: Curr Oncol. 2026 Jun 25;33(7):386. doi: 10.3390/curroncol33070386 (PMC13408374; doi:10.3390/curroncol33070386)
Supplement: Supplementary file 1 [file curroncol-33-00386-s001.zip › curroncol-4326688-supplementary.pdf]

Table S1: PRISMA checklist

Section and Topic	Item #	Checklist item	Location where item is reported
**TITLE**			
Title	1	Identify the report as a systematic review.	Title
**ABSTRACT**			
Abstract	2	See the PRISMA 2020 for Abstracts checklist.	Abstract
**INTRODUCTION**			
Rationale	3	Describe the rationale for the review in the context of existing knowledge.	Introduction, paragraph 4-6
Objectives	4	Provide an explicit statement of the objective(s) or question(s) the review addresses.	Introduction, final paragraph
**METHODS**			
Eligibility criteria	5	Specify the inclusion and exclusion criteria for the review and how studies were grouped for the syntheses.	Eligibility criteria, PICO
Information sources	6	Specify all databases, registers, websites, organisations, reference lists and other sources searched or consulted to identify studies. Specify the date when each source was last searched or consulted.	Information sources and search strategy
Search strategy	7	Present the full search strategies for all databases, registers and websites, including any filters and limits used.	Information sources and search strategy
Selection process	8	Specify the methods used to decide whether a study met the inclusion criteria of the review, including how many reviewers screened each record and each report retrieved, whether they worked independently, and if applicable, details of automation tools used in the process.	Study selection
Data collection process	9	Specify the methods used to collect data from reports, including how many reviewers collected data from each report, whether they worked independently, any processes for obtaining or confirming data from study investigators, and if applicable, details of automation tools used in the process.	Data extraction
Data items	10a	List and define all outcomes for which data were sought. Specify whether all results that were compatible with each outcome domain in each study were sought (e.g. for all measures, time points, analyses), and if not, the methods used to decide which results to collect.	Eligibility criteria, PICO
	10b	List and define all other variables for which data were sought (e.g. participant and intervention characteristics, funding sources). Describe any assumptions made about any missing or unclear information.	Data extraction
Study risk of bias assessment	11	Specify the methods used to assess risk of bias in the included studies, including details of the tool(s) used, how many reviewers assessed each study and whether they worked independently, and if applicable, details of automation tools used in the process.	Risk of bias assessment
Effect measures	12	Specify for each outcome the effect measure(s) (e.g. risk ratio, mean difference) used in the synthesis or presentation of results.	Synthesis and data analysis
Synthesis	13a	Describe the processes used to decide which studies were eligible for each synthesis (e.g. tabulating the study intervention characteristics and	Synthesis

Section and Topic	Item #	Checklist item	Location where item is reported
methods		comparing against the planned groups for each synthesis (item #5)).	and data analysis
	13b	Describe any methods required to prepare the data for presentation or synthesis, such as handling of missing summary statistics, or data conversions.	Synthesis and data analysis
	13c	Describe any methods used to tabulate or visually display results of individual studies and syntheses.	Reporting guidelines
	13d	Describe any methods used to synthesize results and provide a rationale for the choice(s). If meta-analysis was performed, describe the model(s), method(s) to identify the presence and extent of statistical heterogeneity, and software package(s) used.	Synthesis and data analysis
	13e	Describe any methods used to explore possible causes of heterogeneity among study results (e.g. subgroup analysis, meta-regression).	Reporting guideline
	13f	Describe any sensitivity analyses conducted to assess robustness of the synthesized results.	NA
Reporting bias assessment	14	Describe any methods used to assess risk of bias due to missing results in a synthesis (arising from reporting biases).	Reporting bias assessment
Certainty assessment	15	Describe any methods used to assess certainty (or confidence) in the body of evidence for an outcome.	Certainty of evidence
**RESULTS**			
Study selection	16a	Describe the results of the search and selection process, from the number of records identified in the search to the number of studies included in the review, ideally using a flow diagram.	Study selection figure 1
	16b	Cite studies that might appear to meet the inclusion criteria, but which were excluded, and explain why they were excluded.	Figure 1
Study characteristics	17	Cite each included study and present its characteristics.	Table 2
Risk of bias in studies	18	Present assessments of risk of bias for each included study.	Table 4
Results of individual studies	19	For all outcomes, present, for each study: (a) summary statistics for each group (where appropriate) and (b) an effect estimate and its precision (e.g. confidence/credible interval), ideally using structured tables or plots.	Table 3
Results of syntheses	20a	For each synthesis, briefly summarise the characteristics and risk of bias among contributing studies.	Narrative synthesis
	20b	Present results of all statistical syntheses conducted. If meta-analysis was done, present for each the summary estimate and its precision (e.g. confidence/credible interval) and measures of statistical heterogeneity. If comparing groups, describe the direction of the effect.	Narrative synthesis
	20c	Present results of all investigations of possible causes of heterogeneity among study results.	Narrative synthesis
	20d	Present results of all sensitivity analyses conducted to assess the robustness of the synthesized results.	NA
Reporting biases	21	Present assessments of risk of bias due to missing results (arising from reporting biases) for each synthesis assessed.	Reporting bias

Section and Topic	Item #	Checklist item	Location where item is reported
Certainty of evidence	22	Present assessments of certainty (or confidence) in the body of evidence for each outcome assessed.	NA
**DISCUSSION**			
Discussion	23a	Provide a general interpretation of the results in the context of other evidence.	Discussion summary
	23b	Discuss any limitations of the evidence included in the review.	Discussion limitation
	23c	Discuss any limitations of the review processes used.	Discussion limitation
	23d	Discuss implications of the results for practice, policy, and future research.	Discussion – clinical and research implication
**OTHER INFORMATION**			
Registration and protocol	24a	Provide registration information for the review, including register name and registration number, or state that the review was not registered.	Methods study design
	24b	Indicate where the review protocol can be accessed, or state that a protocol was not prepared.	Methods study design
	24c	Describe and explain any amendments to information provided at registration or in the protocol.	NA
Support	25	Describe sources of financial or non-financial support for the review, and the role of the funders or sponsors in the review.	Funding statement
Competing interests	26	Declare any competing interests of review authors.	Conflicted of interest
Availability of data, code and other materials	27	Report which of the following are publicly available and where they can be found: template data collection forms; data extracted from included studies; data used for all analyses; analytic code; any other materials used in the review.	Data availability statement

Table S2 Search string strategy

Database	Full Search String / Fields	Filters Applied	Records Retrieved
PubMed / MEDLINE	(("Neoplasms"[MeSH Terms] OR "neoplasm"[tiab] OR "cancer"[tiab] OR "carcinoma"[tiab] OR "tumor"[tiab] OR "tumour"[tiab] OR "malignancy"[tiab] OR	Humans; Adults (≥18 years); English language; Article type: randomized controlled trial / clinical trial; No date limit (inception–Jan 2025)	195

	"oncology"[tiab]) AND ("Antineoplastic Agents"[MeSH Terms] OR "chemotherapy"[tiab] OR "systemic therapy"[tiab] OR "systemic anticancer therapy"[tiab] OR "targeted therapy"[tiab] OR "immunotherapy"[tiab] OR "endocrine therapy"[tiab] OR "hormonal therapy"[tiab] OR "oral anticancer"[tiab]) AND ("Telemedicine"[MeSH Terms] OR "telehealth"[tiab] OR "telemedicine"[tiab] OR "mHealth"[tiab] OR "m-health"[tiab] OR "eHealth"[tiab] OR "e-health"[tiab] OR "mobile health"[tiab] OR "mobile application"[tiab] OR "smartphone app"[tiab] OR "web-based"[tiab] OR "web portal"[tiab] OR "interactive voice response"[tiab] OR "IVR"[tiab] OR "videoconferencing"[tiab] OR "teleconsultation"[tiab] OR "remote monitoring"[tiab] OR "digital health"[tiab] OR "electronic patient-reported outcome"[tiab] OR "ePRO"[tiab]) AND ("Nursing"[MeSH Terms] OR "nurses"[MeSH Terms] OR "nurse-led"[tiab] OR "nursing intervention"[tiab] OR "nurse practitioner"[tiab] OR "advanced practice nurse"[tiab] OR "nurse navigator"[tiab] OR "oncology nurse"[tiab] OR "nursing staff"[tiab] OR "nursing professional"[tiab]) AND ("Randomized Controlled Trial"[pt] OR "randomized controlled trial"[tiab] OR "RCT"[tiab] OR "random allocation"[MeSH Terms] OR "randomly assigned"[tiab] OR "clinical trial"[pt]))		
Scopus	TITLE-ABS-KEY ((neoplasm OR cancer OR carcinoma OR tumor OR tumour OR malignancy OR oncology) AND (chemotherapy OR "systemic therapy" OR "systemic anticancer" OR "targeted therapy" OR immunotherapy OR "endocrine therapy" OR "oral anticancer") AND (telehealth OR telemedicine OR mhealth OR "m-health" OR ehealth OR "e-health" OR "mobile health" OR "mobile application" OR	Document type: article OR review; Language: English; Publication year: 2000–Jan 2025	110

	smartphone OR "web-based" OR "web portal" OR "interactive voice response" OR IVR OR videoconferencing OR "remote monitoring" OR "digital health" OR "electronic patient-reported outcome" OR ePRO) AND ("nurse-led" OR "nursing intervention" OR "nurse practitioner" OR "advanced practice nurse" OR "nurse navigator" OR "oncology nurse" OR "nursing staff") AND ("randomized controlled trial" OR RCT OR "randomised controlled trial" OR "random allocation")) AND DOCTYPE (ar OR re) AND PUBYEAR > 1999		
CINAHL	(MH "Neoplasms+" OR TI (cancer OR carcinoma OR tumor OR malignancy) OR AB (oncology)) AND (MH "Antineoplastic Agents+" OR TI (chemotherapy OR "systemic therapy" OR "targeted therapy" OR immunotherapy OR "endocrine therapy") OR AB ("oral anticancer" OR "systemic anticancer")) AND (MH "Telemedicine+" OR MH "Mobile Applications" OR TI (telehealth OR telemedicine OR mhealth OR "mobile health" OR "web-based" OR "interactive voice response" OR IVR OR videoconferencing OR "digital health" OR ePRO OR "remote monitoring") OR AB (telehealth OR mhealth OR "mobile application" OR smartphone OR "web portal" OR "digital health" OR "electronic patient-reported")) AND (MH "Nurses+" OR MH "Nursing Care+" OR MH "Advanced Practice Nursing+" OR TI ("nurse-led" OR "nursing intervention" OR "nurse practitioner" OR "nurse navigator" OR "oncology nurse") OR AB ("nurse-led" OR "nursing professional" OR "nursing staff")) AND (MH "Randomized Controlled Trials" OR PT "Randomized Controlled Trial" OR TI ("randomized controlled trial" OR RCT OR "randomly assigned") OR AB ("random allocation" OR "randomly allocated" OR "randomised"))	Age group: adult; Language: English; Publication type: academic journal; Research article; Year: inception–Jan 2025	75

ScienceDirect	("cancer" OR "neoplasm" OR "oncology" OR "tumor") AND ("chemotherapy" OR "systemic therapy" OR "targeted therapy" OR "immunotherapy" OR "endocrine therapy") AND ("telehealth" OR "telemedicine" OR "mHealth" OR "mobile health" OR "mobile application" OR "web-based" OR "interactive voice response" OR "digital health" OR "remote monitoring" OR "electronic patient-reported outcome") AND ("nurse-led" OR "nursing intervention" OR "nurse practitioner" OR "nurse navigator" OR "oncology nurse") AND ("randomized controlled trial" OR "RCT" OR "random allocation")	Article type: research articles; Subject area limited to health sciences / nursing / medicine; Language: English; Year: inception–Jan 2025	90
Total			470
